# Miscellany

**Published:** 1895-12

**Authors:** 


### Miscellany.

REMOVAL.—Dr. Charles Sherman Jewett, of Buffalo, has removed his office and residence from 153 Woodlawn avenue to 892 Main street. Hours: 8–10 A. M., 2–3 and 7 P. M. Sundays, 8–10 A. M., 3–4 P. M.

---

ST. LUKE'S HOSPITAL, New York, is about to obtain an endowment for its pathological department in the sum of $200,000. Resolutions looking toward this end were introduced by George Macculloch Miller, president of the board of managers, at a recent meeting. Responses came so prompt that Mr. Miller is quoted as saying that the desired endowment is already a fact actually accomplished.

